# Voriconazole-induced Myositis in a Double Lung Transplant Recipient

**DOI:** 10.7759/cureus.3998

**Published:** 2019-02-01

**Authors:** Mohanad Soliman, Olalekan Akanbi, Cameron Harding, Mohamed EL-Helw, Michael Anstead

**Affiliations:** 1 Internal Medicine, University of Kentucky School of Medicine, Lexington, USA; 2 Cardiology, University of Kentucky School of Medicine, Lexington, USA

**Keywords:** myositis, myopathy, toxic, prophylaxis, antifungal safety profile, mri musculoskeletal

## Abstract

Voriconazole is a triazole antifungal agent commercially approved in 2002. It is commonly used in immunocompromised patients as a therapeutic and prophylactic agent. We present the case of a 26-year-old Caucasian female who is a double lung transplant recipient who presented with complaints of generalized left lower extremity swelling and extreme tenderness of her left thigh. Although her muscle enzymes were not significantly elevated, inflammatory changes were noticed on T2-weighted fat-suppressed short-TI inversion recovery (STIR) sequence magnetic resonance imaging (MRI). These findings were later confirmed with tissue biopsy. We hereby present the case of drug-induced myositis as a rare complication of voriconazole used as chemoprophylaxis in a double lung transplant recipient patient.

## Introduction

Voriconazole is a triazole antifungal approved by the Food and Drug Administration in 2002 for the treatment of invasive aspergillosis [1]. Metabolized in the liver with excellent oral bioavailability, it exerts its effect by inhibiting ergosterol biosynthesis, a vital component of the fungal cell wall [1, 2]. Although well tolerated, commonly reported and established adverse effects are visual disturbances, liver function abnormalities, and rash which may warrant discontinuation of therapy [1]. Cutaneous malignancies, arrhythmias, neurologic toxicity, alopecia, nail changes, and electrolyte abnormalities are some of its recently reported and more rare adverse effects [3]. Voriconazole-related myopathy has been rarely reported. To the best of our knowledge, only one such case has been described in the literature [4]. We report a case of voriconazole-associated myopathy with associated imaging and histopathologic changes that improved upon drug discontinuation.

## Case presentation

A 26-year-old Caucasian female with a past medical history of bilateral lung transplant for cystic fibrosis, end-stage renal disease on hemodialysis, pancreatic insufficiency, diabetes mellitus, hypothyroidism, hypertension, insomnia, seizures, and chronic pain presented with complaints of chest pain for one day along with a left lower extremity painful swelling that started four days prior to presentation. The chest pain was left-sided, sharp, non-radiating, with no aggravating or relieving factors. Her left lower extremity swelling extended from the hip down to her foot with tenderness most pronounced in the left thigh. Classic symptoms/signs of hypothyroidism such as lethargy, cold intolerance, myxedematous facies, constipation, and/or bradycardia were absent. Her daily home medications included tacrolimus, prednisone 5 mg daily, voriconazole 200 mg every 12 hours, azithromycin, amlodipine, pantoprazole, levothyroxine, pancrelipase (CREON), oxcarbazepine, amitriptyline, gabapentin, sevelamer carbonate, cetirizine, montelukast, and correctional sliding scale insulin.

Investigation

On examination she was in pain, cachectic, and in no acute respiratory distress. She weighed 46 kilograms (kg) with a BMI of 15.3. Her blood pressure was 171/101 mmHg, respiratory rate 18, heart rate 90, temperature 98 F, and oxygen saturation 96% on 2 liters (L) nasal cannula oxygen. She had temporal and masseter muscles wasting, with moist mucous membranes, with no oral thrush or ulcers. On pulmonary auscultation, there was diminished air entry bilaterally. Her abdominal and cardiovascular examinations were unremarkable. Her lower extremities both had pitting edema but it was worse on the left. There was marked tenderness of the posterior left thigh with mild left calf tenderness. She had intact pulses bilaterally with no skin discoloration or darkening of the toes.

**Figure 1:**
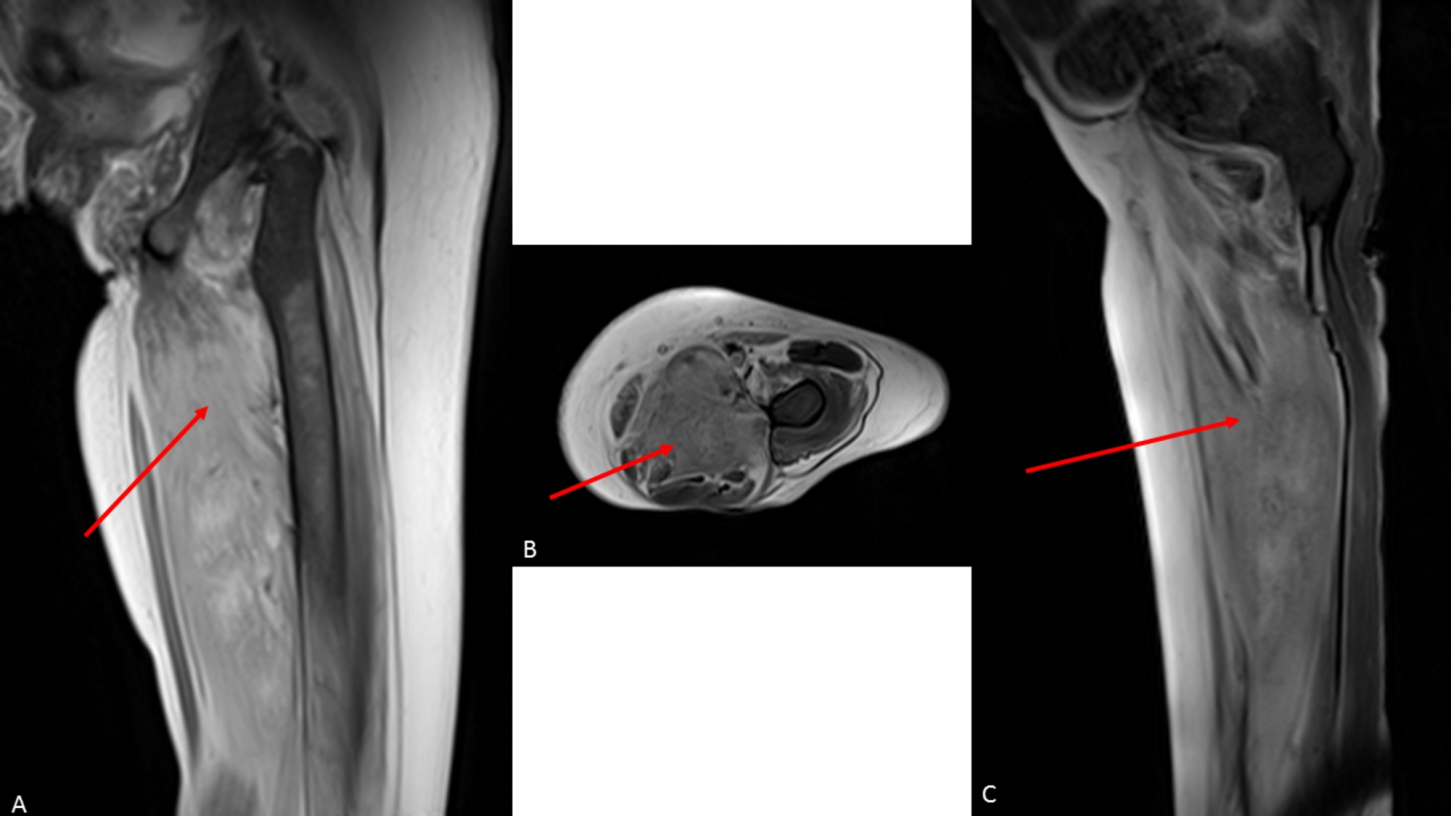
(A) MRI STIR coronal image, (B) MRI T2 fat saturated axial image, and (C) MRI T2 fat saturated sagittal image demonstrating diffuse subcutaneous edema and edema in thigh musculature that was most pronounced within the hamstring muscles (red arrows). MRI - magnetic resonance imaging, STIR - short-TI inversion recovery

Bilateral lower extremity Doppler and chest computed tomography with intravenous contrast pulmonary embolism (PE) protocol scan showed no deep venous thrombosis or PE. Troponin T was mildly elevated with no delta and no evidence of ischemia on the electrocardiogram (ECG). Her labs showed a normal creatinine phosphokinase (CPK) level of 27 U/L, mildly elevated serum aldolase of 8.7 U/L, thyroid-stimulating hormone (TSH) of 11.42 mIU/ml, C-reactive protein of 1.5 mg/dL, lactate dehydrogenase of 204 U/L, serum creatinine of 4.3 mg/dl, blood urea nitrogen of 44 mg/dl, 25 hydroxyvitamin D of 24 ng/ml, HbA1c of 5.1%, serum IgE level of less than 2 KU/L, tacrolimus serum level of 7.6 ng/ml, and white blood cell count of 3 k/uL. Serology was negative for anti-Sm and anti-RNP. A viral polymerase chain reaction (PCR) test for human immunodeficiency virus (HIV), herpes simplex virus (HSV), cytomegalovirus (CMV), Epstein-Barr (EBV) was negative. Her aspergillus galactomannan, serum histoplasma antigen, serum cryptococcal antigen were negative with positive beta-glucan. Radiograph of the left femur showed no skeletal abnormalities. With the persistence of her left leg pain, magnetic resonance imaging (MRI) without intravenous contrast was obtained to evaluate her soft tissue. MRI results demonstrated diffuse subcutaneous edema and edema in thigh musculature that was most pronounced within the hamstring muscles (red arrow). These findings were most consistent with myositis (Figure 1).

**Figure 2:**
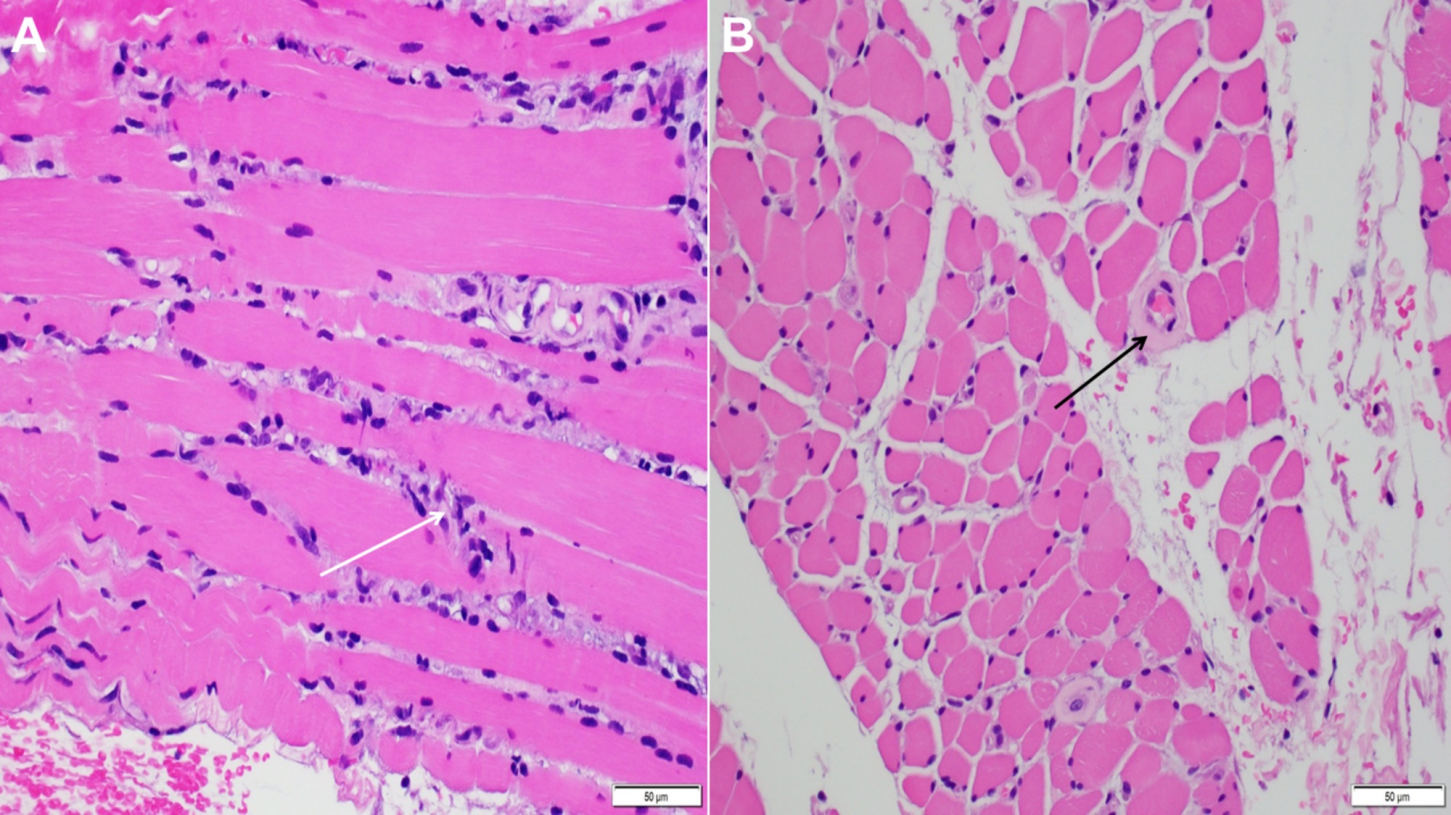
Left thigh skeletal muscle biopsy: image A) degenerating myofibers with macrophage infiltration (white arrow), image B) thickened blood vessels (black arrow) and Congo Red stain is negative for amyloid.

Muscle biopsy was subsequently performed for further assessment and refining the myopathy subtype. It showed A) degenerating myofibers with macrophage infiltration (white arrow). This morphologic appearance typically favors a toxic/drug-induced myopathic process, B) arterioles appear somewhat thickened and hyalinized (black arrow) that warranted a Congo red stain, and the Congo Red stain was negative for amyloid (Figure 2).

Treatment

Muscle biopsy was mostly consistent with drug-induced myositis. Upon reviewing her home medications for any potential causative agent, voriconazole was recognized as a possible inciting factor of her myositis. Her voriconazole was discontinued, and her prednisone dose was increased from 5 mg daily to 40 mg daily. She was slowly tapered by 5 mg every five days and continued on 10 mg after that to help with muscle swelling and inflammation. The patient experienced clinical improvement in terms of reduced left lower extremity swelling and resolution of pain and tenderness of the left thigh on her follow-up visit as an outpatient. Documentation of this adverse drug reaction was made in her chart for references. Of note, as her TSH was slightly elevated, free T4 was checked during the follow-up visit and was found to be 0.6 ng/dl. A higher dose of Synthroid was prescribed and three months later her TSH was 3.7 uIU/ml.

## Discussion

Drug-induced myopathy (toxic myopathy) is the acute or subacute development of myopathic symptoms such as muscle weakness, swelling, myalgias, CPK elevation, or myoglobinuria after being exposed to certain drugs. It usually develops in patients without a prior history of muscle pathology. It has been classified according to the presence or absence of muscular pain into painful and painless toxic myopathies. Painful myopathies can be seen with statins, cimetidine, zidovudine, clofibrate, and cyclosporine. Painless myopathies would be more typical with corticosteroids, colchicine, chloroquine, hydroxychloroquine, antibiotics, and beta-blockers [5].

Drug-induced myopathy may occur through different mechanisms: a) direct muscle toxicity includes alcohol, cocaine, glucocorticoids, lipid-lowering drugs, colchicine, and antiviral zidovudine, b) immune-mediated inflammatory response as in muscle disease associated with D-penicillamine, c) indirect effect on muscle by causing electrolytes imbalance (hypokalemia), hyperthermia, dystonia or coma with ischemic muscle compression [6,7].

Patients must meet several criteria for reporting drug-induced myopathy: a) absence of pre-existing muscular complaints, b) free period between the beginning of the treatment and the appearance of symptoms, c) ruling out other causes of myopathy, d) complete or incomplete improvement after withdrawal of the offending agent [8].

Since the early 1990s, lung transplantation has become an accepted intervention for patients with advanced lung disease [9]. Lung transplant recipients are at an increased risk of infectious complications including a high rate of invasive aspergillosis (IA) with an incidence of 6%-17% and a reported mortality rate of 9% [10]. It is routine for lung transplant centers to employ universal prophylaxis targeting aspergillus species within the first six months post transplant. Voriconazole alone and in combination with inhaled amphotericin B (AmB) are the preferred first‐line agents [11]. Our patient was started on voriconazole as a chemoprophylactic agent as she was receiving different cycles of antibiotics for recurrent episodes of pneumonia.

MRI has been shown to be an acceptable modality in evaluating muscle disorders. Not only can it help establish the diagnosis of myositis but it can aid in refining the myositis phenotype and to monitor response to therapy. Although muscle biopsy is the gold standard diagnostic modality, MRI can decrease the rate of false negatives by directing biopsy site selection [12].

It has been previously demonstrated that significant myopathies confirmed by muscle biopsy can develop in the setting of normal CPK levels. It has been suggested that CPK is an inadequate test for statin-induced myopathy [13]. It was also reported in a case series that inflammatory myopathies could present with normal CPK. In these cases, it was associated with a poor prognosis [9].

## Conclusions

As voriconazole becomes the standard of care as a prophylactic and therapeutic agent in immunosuppressed and transplant recipients, one must be clinically aware of its potential adverse effects. It is essential to recognize it as a possible cause of myositis, particularly in patients with a prior history of drug-induced myopathy. Although severe forms of the resulting myositis are uncommon, milder forms may be more frequent than is generally appreciated. Because drug-induced myopathies are potentially reversible, their early recognition may prevent the clinical consequences that range from a mild muscle ache to profound myalgia and paralysis.
